# A new risk score for patients after first recurrence of stage 4 neuroblastoma aged ≥18 months at first diagnosis

**DOI:** 10.1002/cam4.2562

**Published:** 2019-10-20

**Authors:** Kiana Kreitz, Angela Ernst, René Schmidt, Thorsten Simon, Matthias Fischer, Ruth Volland, Barbara Hero, Frank Berthold

**Affiliations:** ^1^ Institute of Medical Statistics and Clinical Research University of Muenster Muenster Germany; ^2^ Institute of Medical Statistics and Computational Biology University of Cologne Cologne Germany; ^3^ Department of Pediatric Oncology and Hematology University of Cologne Cologne Germany

**Keywords:** clinical trial, high‐risk neuroblastoma, recurrence, relapse, risk score, time‐dependent variable

## Abstract

**Background:**

The prognosis of patients with recurrences from stage 4 neuroblastoma is not uniformly dismal. The evaluation of new therapies therefore needs to consider the individual risks of the treated patients. This study aims to define clinically useful risk criteria.

**Patients and Methods:**

Inclusion criteria were: first recurrence of neuroblastoma stage 4 aged ≥18 months and enrollment in first line trials between 1997 and 2016. Patients were randomized into a training set (N = 310) and an independent validation set (N = 159). The primary endpoint was secondary event‐free survival. The individual treatment elements the patients received during initial and recurrent disease were analyzed as binary and time‐dependent variables. A five‐step multiple time‐dependent Cox regression analysis was performed on the training set to identify prognostic variables adjusted for the individual frontline treatment. The selected variables resulted in a prognostic index (PI) and were used to build a risk score system. The score was validated with the validation set.

**Results:**

Of the 469 patients, 372 were treated with curative intent and 97 with palliative intent. The PI included the variables number of recurrence organs (hazard ratio [HR] = 2.27), time to recurrence (HR = 2.03), liver metastasis at diagnosis (HR = 1.77), first recurrence at site of the primary tumor (HR = 1.55), and age (HR = 1.29). Three risk groups were built and confirmed in the validation set. The scoring system was likewise useful for the curatively or palliatively treated subgroups.

**Conclusion:**

A new risk score system for patients with first recurrence of stage 4 neuroblastoma aged ≥18 months at diagnosis is proposed.

## Graphical abstract

The prognostic index of each patient with first recurrence of stage 4 neuroblastoma aged >18 months at first diagnosis can be calculated. If a characteristic is present, use the indicated figure. If absent, use 0. The sum indicates the related risk group.

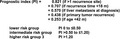

## 1 INTRODUCTION

The prognosis of children with recurrences of high‐risk neuroblastoma is dismal.^1^, ^2^, ^3^, ^4^, ^5^, ^6^, ^7^, ^8^, ^9^ The reported time periods from the observation of first recurrence to the subsequent progression are short (median intervals 58 days,^1^ 4.7 months,^2^ 6.4 months^3^), and the overall survival proportions have been poor (20% after 4 years,^1^ 20% after 5 years,^4^ 7% after 10 years^5^). Risk factors for an inferior outcome included stage 4,^4^, ^5^ age ≥18 months at first diagnosis,^4^, ^5^ MYCN amplification,^1^, ^2^, ^4^, ^6^, ^7^ loss of heterozygosity of chromosome 11q,^1^ shorter time from diagnosis to first recurrence,^4^, ^7^ abdominal primary tumor,^5^ bone marrow metastasis at first diagnosis,^2^ recurrent disease (vs refractory disease),^3^, ^8^, ^10^ and increased lactate dehydrogenase blood levels.^5^ Other predictors for poor outcome were measurable tumor on computed tomography/ magnetic resonance imaging at second‐line trial enrollment, high Curie score by metaiodobenzylguanidine scintigraphy, and stem cell supported therapy.^10^ The analyzed cohorts varied and included recurrences from localized tumors,^1^, ^2^, ^4^, ^5^, ^6^, ^7^, ^8^, ^10^ patients aged less than 18 months at diagnosis,^1^, ^2^, ^4^, ^5^, ^9^, ^10^ and patients with refractory disease.^3^, ^8^, ^10^

For parents, the decision to undergo therapy a second time after recurrence of stage 4 neuroblastoma is heavily dependent on the potential survival chances. For clinical scientists, reporting on phase 2/3 trials requires a comparison with equivalent risk groups. The study objective was to develop a robust score system taking into account the individual treatment the patients had received. A large data set was retrospectively analyzed, focusing on the largest and worst group, ie stage 4 neuroblastoma aged ≥18 months at initial diagnosis, including patients treated with palliative intent.

## 2 MATERIALS AND METHODS

### 2.1 Study design

This is a retrospective analysis of data obtained from the national trials NB97 and NB2004 of the German Pediatric Oncology Society. The data lock was 30 April 2019. The primary endpoint for this analysis was secondary event‐free survival (secEFS), and the secondary endpoint was secondary overall survival (secOS). Table S2 lists the non‐time‐dependent variables used for the analysis.

The trials were conducted in 66 pediatric oncology university and community hospitals in Germany and Switzerland. All protocols were approved by the ethics committees of the University of Cologne. Written informed consent was obtained from the parents or legal guardians before enrollment and included the follow‐up after recurrences until death. Yearly routine check‐up reports were requested by the trial office from the local institutions and/or by the German Childhood Cancer Registry (http://www.kinderkrebsregister.de). If medical care had been finished, the local registration offices were approached in accordance with the national law. Of all German neuroblastoma patients known to the German Childhood Cancer Registry, 99.3% participated in one of the two trials between 1997 and 2016.^11^ High‐dose chemotherapy with autologous blood stem rescue and anti‐GD2 immunotherapy was used systematically from 1997 onwards. Palliative therapy was assumed if the medical report clearly stated the intent of the therapeutic measure was palliative. The decision for palliative or curative treatment was made exclusively at the treating institution. Epidemiological, diagnostic, and therapeutic details are described elsewhere.^11^ One hundred and eighty three study patients have been the subject of an earlier report.^7^

### 2.2 Participants

The inclusion criteria were (a) neuroblastoma stage 4 according to international criteria (INSS)^12^; (b) age at diagnosis ≥18 months and <21 years; (c) diagnosis between 5 March 1997 (trial NB97 first patient in), and 31 December 2016 (trial NB2004 last patient in); (d) enrollment in the national neuroblastoma trials NB97 or NB2004; (e) first recurrence diagnosed. Exclusion criteria were (a) death due to the tumor or early progression <90 days without response to first line therapy; (b) death due to toxicity in first line therapy; (c) no or inadequate first line therapy; (d) diagnosis of a second malignancy at any time; (e) insufficient information regarding recurrence site, recurrence therapy or unknown cause of death in first line therapy.

**Figure 1:**
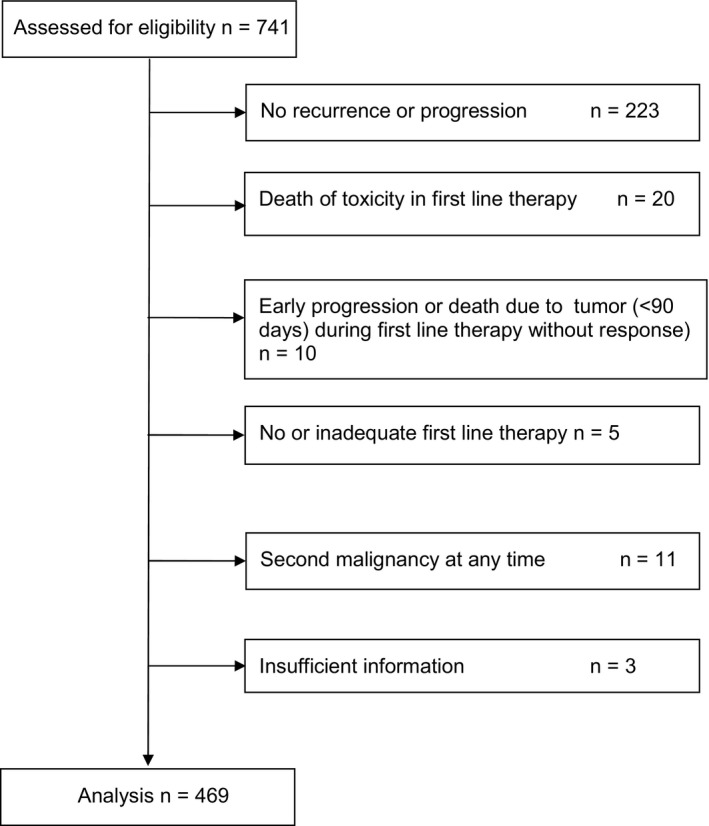
Trial design (CONSORT diagram). Inclusion criteria: neuroblastoma stage 4, diagnosis 5 March 1997 to 31 December 2016, age at diagnosis ≥ 18 months to <21 years, first recurrence

### 2.3 Statistical analysis methods

Secondary event‐free survival was defined as the time from first recurrence until second recurrence or until death of any cause or until the last examination. For simplicity, recurrences where tumors had completely disappeared as well as progressions where tumors had partially disappeared or had been stable have been comprised under the term “recurrences”. Secondary overall survival was defined as the time from first recurrence until death of any cause or until the last examination. Kaplan‐Meier estimates for secEFS and secOS were compared using the log‐rank test. For multivariable survival analyses, the Cox regression model was used. Estimated hazard ratios (HR) with 95%‐confidence intervals (95%‐CI) and Wald *P*‐values were calculated. For all analyses, IBM SPSS statistical package version 24 and SAS version 9.4 were used.

### 2.4 Explanatory variables and missing data

Continuous variables (eg age and time from diagnosis to first recurrence) were entered in continuous and in binary form (median as cut‐off) into the model. Count variables (eg number of recurrence sites) were additionally used in the binary form (one vs more than one). Missing data were treated as missing completely at random. First line therapies were included as binary variables, and therapies with curative intent after the first recurrence were included as time‐dependent variables (effective during treatment time and the following month). Palliative treatment was considered as a binary variable.

### 2.5 Analysis sets

Patients were stratified according to the variables palliative treatment (yes/no), MYCN amplification (yes/no),^1^, ^2^, ^4^, ^6^, ^7^ time from diagnosis to first recurrence (≤18/>18 months),^4^, ^7^ and number of recurrence organs (1/>1) and then randomized with allocation ratio 2:1 into a training set (n = 310) and a validation set (n = 159). All patients treated with palliative (n = 97) or curative (n = 372) intent were included in the analysis in order to obtain the full spectrum.

### 2.6 Score building/development

A multiple time‐dependent Cox regression analysis was performed on the training set to identify prognostic variables that are available at the time of first recurrence diagnosis. As some of the considered variables had missing values and regression analysis is dependent on complete information, variables were categorized into three groups: variables with a high number of missing values (≥15%), variables with some missing values (>0‐<15%) and the variables without any missing data.

**Figure 2:**
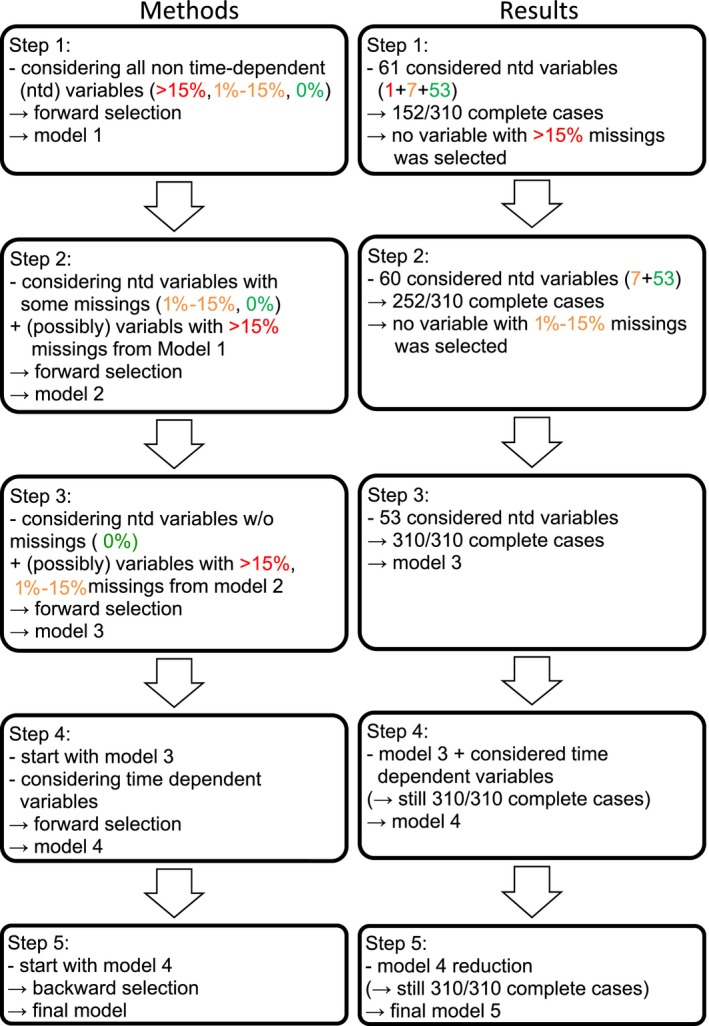
The 5 step variable selection procedure: criteria and resulting number of patients. ntd, non‐time dependent

A 5‐step approach for the selection of variables was used based on consecutive forward selection (inclusion criterion: *P*‐value of score test ≤.05) and backward selection steps (exclusion criterion: *P*‐value of likelihood ratio test >.05). The procedure is summarized in Figure 2.

The first two steps were used to test whether any variables with missing values showed to be significant. In the first step (forward selection), all variables were entered into the model to identify significant variables with ≥15% missing values. The second step of forward selection included variables with <15% missing values plus the variables with ≥15% missing values selected in the first step. This was done in order to have a higher number of complete cases available for analysis. The final model was built in steps three to five. In the third step, only variables without missing values plus the selected variables from the previous step were considered and again chosen through forward selection. In the fourth step, the model from the previous step was expanded by successively adding the treatments as time‐dependent covariates into the forward selection model in order to adjust for the therapy the patients received. Finally, in the fifth step the set of variables was reduced via backward selection, resulting in the final model

(1) $$\text{Hazard}\left(t,\;X,\;Y,\;Z\right)=h_{0}\left(t\right)\cdot exp\left(X\beta \right)\cdot exp\left(Y\gamma \right)\cdot exp\left(Z\left(t\right)\delta \right),$$

where *X* is the design matrix of the nontherapy variables, *Y* is the design matrix of non‐time‐dependent therapy variables, and *Z*(*t*) contains the time‐dependent (second‐line therapy) variables. The vectors *β*, *γ*, and *δ* depict the corresponding vectors of estimated regression coefficients. The model fit was assessed by estimating the integrated time‐dependent area under the curve and Harrell's concordance statistic.

### 2.7 Prognostic index and risk score

The parameter estimates of the selected nontherapy variables were then used to produce the prognostic index (PI), PI = *Xβ*. The PI is a measure of the risk for an event independent of the received therapy. It can be calculated for each patient based on his/her characteristics regarding the prognostic variables. Based on an optimal stratification of the PI, three risk groups were built. This was done using all possible pairs of cut‐off values. Each cut‐off pair defined three risk groups and was evaluated in a Cox regression model including the risk groups plus the therapeutic variables of the final model. Finally, the cut‐off pair with the smallest *P*‐value in the likelihood ratio test was chosen (unadjusted for the multiple tests of all possible cut‐off values).

The resulting risk groups defined the risk score.

### 2.8 Score validation

The risk score was applied to the independent validation set in order to test the reliability to discriminate the three subgroups regarding secEFS.

The overall confirmatory null hypothesis was:

H0: the secEFS of patients in the lower risk group is equal to or worse than the secEFS of patients in the intermediate risk group or the secEFS of patients in the higher risk group is equal to or better than the secEFS of patients in the intermediate risk group.

The *P*‐value is calculated as *P*:= max[*P1,P2*] where *P*1 [*P*2] is the one‐sided Wald *P*‐value of the variable risk group (higher [lower] risk group vs intermediate risk group) in the Cox regression model including risk group and the therapy variables from the last step of model building. The same hypotheses were exploratively evaluated for secOS. To investigate the robustness of the score, the analysis was repeated with risk group as the only explanatory variable. The results obtained were exploratively reassessed for the cohort of curatively treated patients. Additionally, the five diagnostic variables from the final model were separately analyzed in a Cox regression model including the therapy variables from the last step.

## 3 RESULTS

Four hundred and sixty nine cases with recurrences of stage 4 neuroblastoma aged ≥18 months at initial diagnosis were identified. The CONSORT diagram (Figure 1) shows the numbers of patients who were excluded from the analysis.

### 3.1 Recurrence sites

Table S1 shows that the most frequently involved recurrence organs were the osteomedullary site and the primary tumor area. In one quarter of the patients, both sites were jointly affected. The recurrence presented in 56.3% of cases in one site/organ, in 30.7% in two, in 10.2% in three, and in 2.8% in four sites.

### 3.2 Survival proportions and times

The 5‐year secEFS of the total group (N = 469) was 8% (95%‐CI 6‐10), and the 5‐year secOS was 10% (95%‐CI 6‐14). The median time from the first to the second event was 5.4 months (95%‐CI 4.4‐6.4) and to death 12.6 months (95%‐CI 10.9‐14.4).

Twenty one percent of patients were treated with palliative intent. The frequency decreased from 32% during 1997‐2004 to 14% during 2004‐2019 (Pearson's *χ* ^2^ test, *P* < .001). Patients treated with palliative intent had a shorter median secEFS time (1.8 months, 95% CI 1.3‐2.3, N = 97) than curatively treated patients (8.0 months, 95% CI 6.2‐9.7; N = 372, *P* < .001). The median secOS times were 2.1 months (95% CI 1.6‐2.6) for palliatively treated patients and 16.1 months (95% CI 13.8‐18.3, *P* < .001) for curatively treated patients. The median time from the first to second event and to death remained stable over time for the patients treated with curative intent (median secEFS times NB97/NB2004 8.6/7.7 months; median secOS times NB97/NB2004 15.7/17.6 months, respectively).

### 3.3 Score building/development

The training set for the Cox regression analysis had 310 cases. Sixty‐one non‐time‐dependent variables were available at the time of diagnosis of the first recurrence, including the variable “palliative treatment” (Table S2). In the first step, all 61 variables were considered for selection, resulting in 152/310 complete cases available. No variable with more than 15% missing values was selected. In the second step, the 60 variables with less than 15% missing values were considered, resulting in 252/310 complete cases. Again, none of the variables with missing data was selected and the whole training set could be used only including the 53 variables without missing data. The model chosen in the third step was then expanded in the fourth step by adding second‐line therapies as time‐dependent variables (Table S3). The final model from the fifth step (backward selection) is depicted in Table 1. All nontherapy parameters are generally available at the time of recurrence and were therefore chosen to build a PI. Aberrations of chromosome 1p and amplification of the MYCN were prognostic in univariable analysis (Table S2), but not relevant in multivariable analysis (Table 1). Lactic dehydrogenase elevation was univariably and multivariably not prognostically relevant. The PI allows the individual risk of each patient to be calculated (Table S4) according to the formula (coefficients rounded):

(2) $$\begin{array}{cc} \text{PI}= & 0.821\;(if\;>1\;\text{recurrence}\;\text{site})+0.707\;(if\;\text{recurrence}\;\text{time}\;<18\;m)\\ & +0.570\;(if\;\text{liver}\;\text{metasis}\;\text{at}\;\text{diagnosis})\\ & +0.438/\;(\mathrm{if}\;\mathrm{primary}\;\mathrm{tumor}\;\mathrm{recurrence})\\ & +0.253\;(\mathrm{if}\;\mathrm{age}\;<42\;m). \end{array}$$

**Table 1:** Multivariable analysis of risk factors for secondary event‐free survival and their prognostic estimate.^a^

Parameter	HR	95% CI		Regression coefficient^c^	±SE	*P*(*χ* ^2^)
Time to first recurrence ≤18 vs >18 mo^b^	2.027	1.558	2.639	0.70675	0.13444	<.0001
Liver metastasis at diagnosis Yes vs No^b^	1.768	1.218	2.565	0.56981	0.18994	.0027
Number of recurrent organs >1 vs 1^b^	2.272	1.698	3.038	0.82050	0.14839	<.0001
Age at diagnosis <42 vs ≥42 mo^b^	1.287	1.009	1.642	0.25263	0.12409	.0418
First recurrence in primary tumor site Yes^b^ vs No	1.549	1.162	2.066	0.43766	0.14680	.0029
Palliative therapy Yes vs No^b^	2.992	2.091	4.283	1.09606	0.18295	<.0001
Treatment with high‐dose chemotherapy with autologous stem cell transplantation in first line therapy Yes^b^ vs No	1.536	1.159	2.036	0.42942	0.14377	.0028
Chemotherapy after recurrence (time‐dependent) Yes vs No b	0.590	0.430	0.809	−0.52726	0.16102	.0011

Note

The first five variables were used for building the risk score. Training set (N = 339).

Abbreviations: 95% CI, 95%‐confidence interval; HR, hazard ratio; mo, months; SCT, myeloablative therapy with stem cell transplantation; SE, standard error.

a Final model of the fifth step.

b Reference.

c Prognostic estimate refers to the estimated values of the regression coefficients *β_i_*.

**Figure 3:**
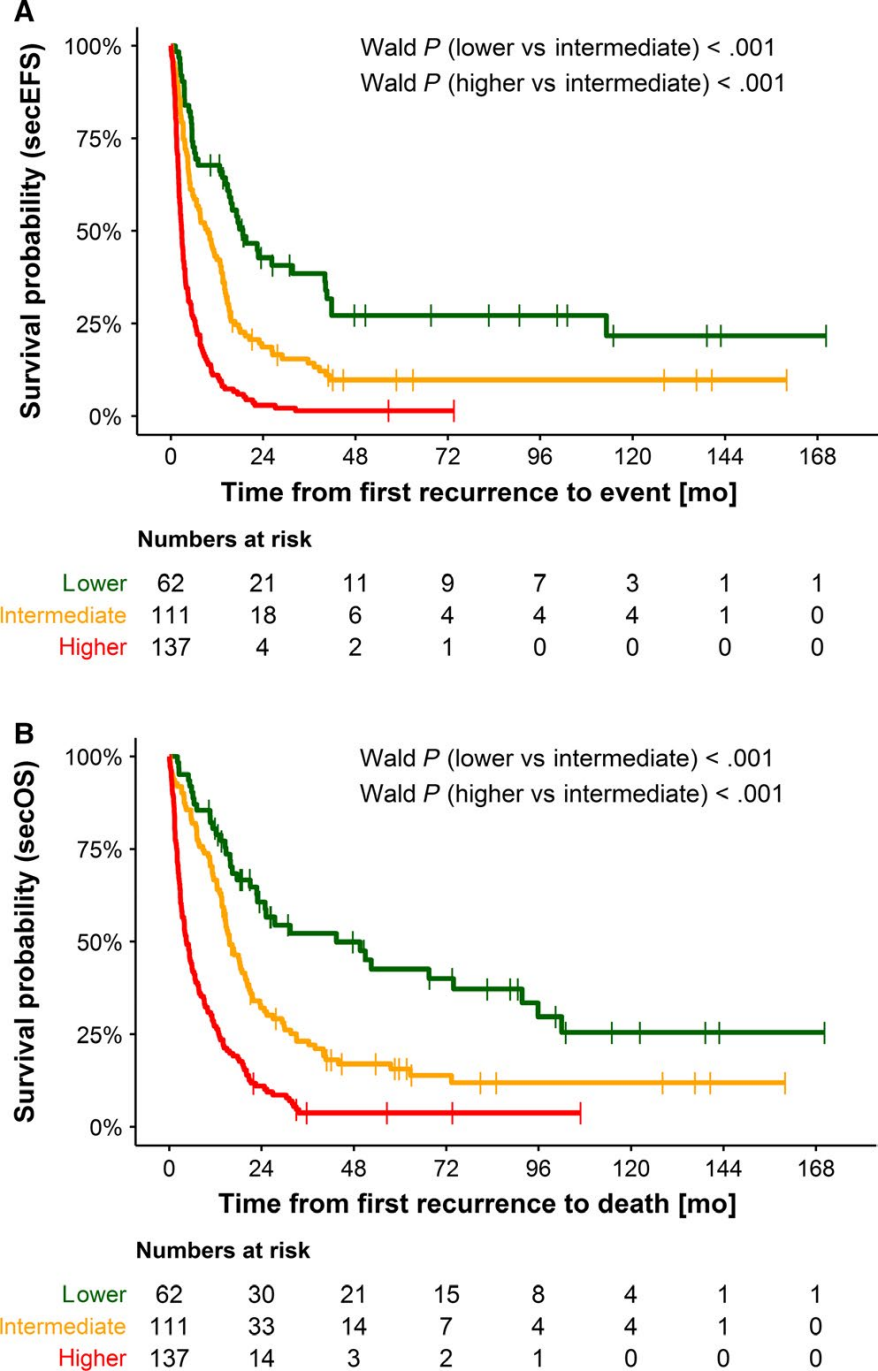
Kaplan‐Meier estimations for secondary event‐free survival (A) and secondary overall survival (B) according to the proposed risk score. Training set n = 330. Risk group lower (n = 62 patients), risk group intermediate (n = 111 patients), and risk group higher (n = 137 patients). A, The 2‐year secEFS proportions were 26.9 % (95% CI 14.6‐39.2) for risk group lower, 9.2% (3.3‐15.1) for risk group intermediate, and 1.7% for risk group higher. B, The 2‐year secOS proportions were 49.3% (95% CI 35.6–63.0) for risk group lower, 16.6% (95% CI 9.2–24.0) for risk group intermediate, and 3.4% (95% CI 0.1–6.7) for risk group higher

Ranging from 0 to 2.787 with a median of 1.11 (95% CI 1.01‐1.14), the optimal two cut‐offs were at *C*_1_ = 0.50 and *C*_2_ = 1.20. Figure 3 shows the Kaplan‐Meier curves of the three risk groups for secEFS in the training set. Twenty percent of patients belonged to risk group 1 (lower, PI 0 to ≤0.50), 36% to risk group 2 (intermediate, PI > 0.50 to ≤1.20), and 44% to risk group 3 (higher, PI > 1.20). The distribution of the identified risk factors for the total group (N = 469) is shown in Table S5.

### 3.4 Score validation

**Figure 4:**
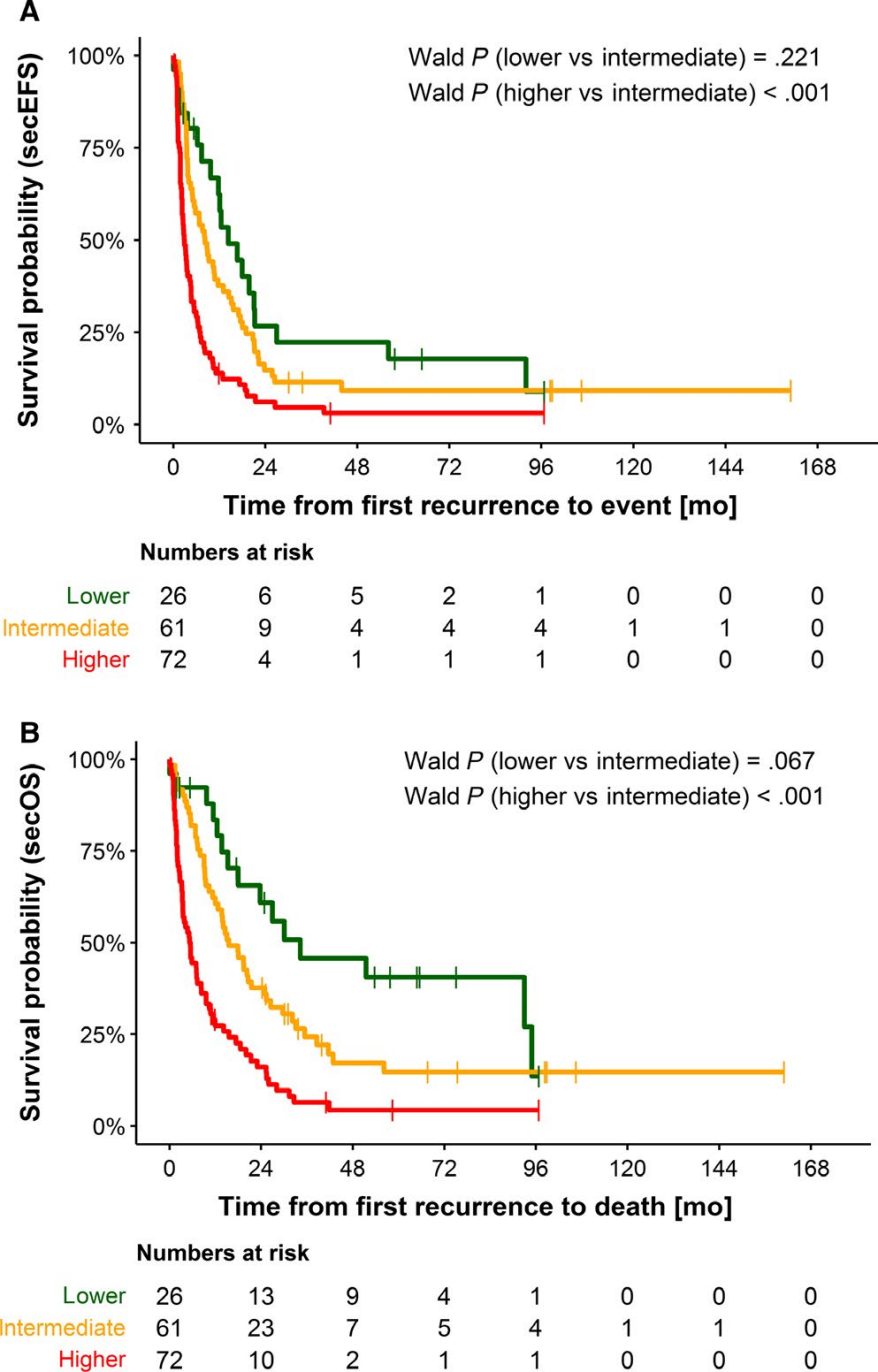
Kaplan‐Meier estimations for secondary event‐free (A) and secondary overall (B) survival according to the proposed risk score. Validation set n = 149. Risk group lower (n = 26 patients), risk group intermediate (n = 61 patients), and risk group higher (n = 72 patients). A, The 2‐year secEFS proportions were 25.5% (95% CI 7.4‐43.6) for risk group lower, 9.2% (95% CI 1.6‐16.8) for risk group intermediate, and 2.7 % (95% CI 0‐6.8) for risk group higher. B, The 2‐year secOS proportions were 47.5% (95% CI 26.2–68.8) for risk group lower, 18.9% (95% CI 8.4–29.3) for risk group intermediate, and 4.3% (95% CI 0–10.3) for risk group higher

The overall null hypotheses that secEFS is independent of risk group could be rejected at a significance level of 2.5% (*P* < .001), thus supporting the validity of the risk score. The validation set consisted of 159 cases, of which 16% were attributed to risk group 1, 38% to risk group 2, and 45% to risk group 3. The risk score reliably discriminated between the groups for secondary event‐free as well as for overall survival. The HRs of the multivariable Cox model for secEFS including the therapeutic variables were 0.82 (95% CI 0.49‐1.38, lower vs intermediate risk, Wald *P* = .462) and 1.97 (95% CI 1.37‐2.83, higher risk vs intermediate risk, *P < *.001). For secOS, the HRs were 0.77 (95% CI 0.43‐1.40, lower vs intermediate risk, *P* = .393) and 2.07 (95% CI 1.41‐3.03, higher vs intermediate risk, *P* < .001). Figure 4 shows the Kaplan‐Meier estimates.

In the subgroup of curatively treated patients (n = 126), the score was different between higher vs intermediate risk as well for secEFS (Wald *P* = .002) and secOS (*P* < .001), while intermediate vs lower risk were not discriminated (secEFS *P* = .253; secOS *P* = .236). In the statistically small subgroup of patients with palliative care (n = 33), the score discriminated also higher risk vs intermediate risk (sec EFS *P* = .016) and lower vs intermediate risk (secEFS *P = *.015, secOS *P* = .046), but not secOS higher vs intermediate risk (*P* = .118). Figure S1 shows the Kaplan‐Meier estimates.

## 4 DISCUSSION

A new scoring system defining three risk groups for patients with recurrences of high‐risk stage 4 neuroblastoma aged ≥18 months is proposed. The data needed for the score are available at the diagnosis of recurrence (age, time to recurrence, liver metastasis at diagnosis, recurrence at the site of the primary tumor, number of recurrence sites).

The strengths of the study are: (a) a focus on a homogeneous group of patients with a poor prognosis (stage 4 aged ≥18 months); (b) the large number of patients analyzed (N = 469); (c) an almost complete coverage of known German patients (99% enrollment into the trials); (d) the inclusion of patients treated with palliative intent; (e) the adjustment for the individual treatment received before the recurrence; (f) the long period of accrual (1997‐2019); (g) and the validation of the score in an independent set of patients.

The study has limitations. One is the retrospective nature of the analysis. The main information was obtained through medical reports and yearly routine queries while other structured detailed reports were only rarely available. Second, the diagnostic work‐up at the time of recurrence was not always complete. For example, bone marrow cytology was rarely performed in the event of osteomedullary metaiodobenzylguanidine accumulation or in cases of palliative treatment intent. Nonetheless, the date and the sites of recurrences used for the analysis were unambiguous. Thus, more recurrence sites were possible, but not fewer. Third, the therapeutic elements applied to treat the recurrence were comprised under one heading, eg “chemotherapy”. Specific data on drugs used, dosages, and timing were rarely available and not analyzed. Thus, only “presence” or “absence” of a therapeutic element and the time period were used for the adjustment. But even this limited information concerning consecutive treatment approaches is rarely available in most data bases. Fourth, the decision to treat a patient curatively or palliatively was up to the patient's family and the local treating team. The information about the therapeutic intent was communicated in all cases from the treating institution. It cannot be excluded that some of the palliative treatment patients may have been categorized as curative intent patients. The portion of patients treated palliatively decreased with time. Fifth, it was not our goal to analyze the effect of different treatments patients received. As the secondary treatment variables are included in the analysis as time‐dependent variables assuming a constant effect of therapy during treatment plus four weeks not considering the doses and other factors, the parameters describing the effect of the treatment must be interpreted with care and we do not suggest any therapy recommendation based on the found effects. Sixth, the designations “lower”, “intermediate”, and “higher” are relative and must be viewed in the context of the generally poor survival of this specific cohort of stage 4 patients with first recurrence. Finally, the risk score is not applicable to patients refractory to or without or with inadequate first line therapy (15/741 = 2%, Figure 2).

The combination of the individual risk factors resulting in a score to predict the individual risk is new. Through the inclusion of the therapeutic variables, the resulting parameters from the multivariable analysis yield the effect of the risk factor when corrected for the treatment the patients received. This is the important point of our study. The five described variables appear to cover the impact of *MYCN* oncogene (*MYCN*) amplification,^1^, ^2^, ^4^, ^6^, ^7^ increased lactate dehydrogenase levels,^5^ and other risk factors mentioned. In the future, novel omics technologies may provide better insights into the molecular mechanism and potentially better explain clinical courses right from the first diagnosis.^13^, ^14^ Tumor material for molecular investigations was not available in most cases of this series. The match of phenotype and genotype is an important task for the future.

The authors of this study see two implications: the estimation of the individual risk of a patient for treatment selection and the presentation of basic risk group data for comparisons with outcomes obtained by new therapeutic approaches. The gold standard to evaluate a new therapy is a randomized clinical trial. This score may help to balance the different risk categories within such a trial.

In conclusion, a new risk score system for patients with first recurrences of stage 4 neuroblastoma aged ≥18 months at diagnosis is proposed.

## CONFLICT OF INTEREST

None declared.
