# Supplementary material for: A new risk score for patients after first recurrence of stage 4 neuroblastoma aged ≥18 months at first diagnosis
Source: Cancer Med. 2019 Oct 20;8(17):7236–43. doi: 10.1002/cam4.2562 (PMC6885891; doi:10.1002/cam4.2562)
Supplement: Supplementary file 1 [file CAM4-8-7236-s001.pdf]

Content of Online-Only

Supplemental Information

Supplemental Table S1

Frequencies of organs involved in 469 patients with recurrences

Supplemental Table S2

Frequencies and univariate prognostic impact of the non-time-dependent variables analyzed

Supplemental Table S3

Analyzed time-dependent variables with Cox proportional hazard estimations for secondary event-free survival

Supplemental Table S4

Distribution of prognostic index values and risk classes in the training set, validation set and total set

Supplemental Table S5

Distribution of the identified risk factors in the defined risk groups.

Supplemental Figure S1

Kaplan-Meier estimations for secondary event-free and secondary overall survival according to the proposed risk score and with curative intent.

**Supplemental Figure S1A**	EFS of patients treated with curative intent
**Supplemental Figure S1B**	OS of patients treated in curative intent
**Supplemental Figure S1C**	EFS of patients treated in palliative intent
**Supplemental Figure S1D**	OS of patients treated in palliative intent

Supplemental Table S1

Frequencies of organs involved in 469 patients with recurrences

**Recurrence site**	**%**	***N****
Primary tumor site	46.7	219
Osteomedullary	66.5	312
Central nervous system	14.5	68
Lymph nodes	10.0	47
Liver	10.4	49
Lung/pleura	4.5	21
Other sites	2.6	12
**special cases of recurrence sites**	**%**	***N****
Primary tumor alone	15.4	72
Osteomedullary alone	27.5	129
Central nervous system alone	9.4	44
Lymph nodes alone	1.3	6
Primary tumor and osteomedullary	27.1	127
Primary tumor and CNS	0.9	4

*number of patients with involved sites

Central nervous system (CNS) metastasis: presence of neuroblastoma cells in cerebrospinal liquor, intracerebral lesions, and/or limited nodular or areal thickening of meninges diagnosed by computed tomography or magnetic resonance imaging.

Osteomedullary metastases: scintigraphic accumulation of the tracer in the skeleton and comprised bone marrow and bone.

Supplemental Table S2

Frequencies and univariate prognostic impact of the non-time-dependent variables analyzed (*N* =469)

Variable	Missings*	Median (Range)	Categories	numbers (percent)	Wald *P*	Comparison	HR (95% CI)
**LOH 1p aberration**	167	n.a.	no (ref.)	150 (50)	0.010	yes vs. no	1.37 (1.08 – 1.74)
			yes	152 (50)			
**HVA**	34	n.a.	normal/borderline for age (ref.)	40 (9)	0.115	abnormal vs. normal/borderline	1.34 (0.93 – 1.92)
			abnormal	395 (91)			
**VMA**	26	n.a.	normal/borderline for age (ref.)	74 (17)	0.497	abnormal vs. normal/borderline	1.10 (0.84 – 1.45)
			abnormal	369 (83)			
**NSE**	45	n.a.	normal/borderline for age (ref.)	6 (1)	0.349	abnormal vs. normal/borderline	1.53 (0.63 – 3.69)
			abnormal	418 (99)			
**MYCN amplification**	48	n.a.	no (ref.)	310 (67)	<0.001	yes vs. no	1.44 (1.17 – 1.77)
			yes	155 (33)			
**remission status after induction therapy, summarized**	21	n.a.	CR/VGPR/PR/MR (ref.)	426 (95)	0.015	NR/progression/recurrence vs. VR/TR/sgTR/gR	1.73 (1.11 – 2.69)
			NR/PROG/REC	22 (5)			
**time period of first recurrence**	4	n.a.	during induction therapy (ref.)	39 (8)			
			after induction therapy	280 (60)	0.108	After induction therapy vs. during induction therapy	0.75 (0.53 – 1.07)
			after first line therapy	146 (31)	<0.001	after first line therapy vs. during induction therapy	0.42 (0.29 – 0.61)
**LDH**	7	n.a .	normal/borderline for age (ref.)	20 (4)	0.071	abnormal vs. normal/borderline	1.59 (0.96 – 2.62)
			abnormal	442 (96)			
**age at diagnosis binary**	0	n.a.	<42 months	240 (51)	0.030	<42m vs. ≥42m	1.24 (1.02 – 1.50)
			≥42 months (ref.)	229 (49)			
**age at diagnosis in months**	0	41.0 (31.0 – 59.4)	n.a.		0.677		0.999 (0.997 – 1.002)

**time from diagnosis to first recurrence binary**	0	n.a.	<18 months	230 (49)	<0.001	<18m vs. ≥18m	2.45 (2.01 – 2.99)
			≥18 months (ref)	239 (51)			
**time from diagnosis to first recurrence in months**	0	18.1 (13.0 - 25.1)	n.a.		<0.001		0.97 (0.96 – 0.98)
**first line therapy antibody**	0	n.a.	no (ref.)	371 (79)	0.282	yes vs. no	0.88 (0.69 – 1.11)
			yes	98 (21)			
**first line therapy ASCT**	0	n.a.	no (ref.)	126 (27)	<0.001	yes vs. no	0.60 (0.49 – 0.75)
			yes	343 (73)			
**first line therapy isotretinoin**	0	n.a.	no (ref.)	179 (38)	<0.001	yes vs. no	0.62 (0.51 – 0.76)
			yes	290 (62)			
**first line therapy, maintenance chemotherapy, number of cycles**	0	0 (0 – 0)	n.a.		0.213		1.04 (0.98 – 1.11)
**first line therapy, maintenance chemotherapy**	0	n.a.	no (ref.)	396 (82)	0.122	yes vs. no	1.23 (0.95 – 1.59)
			yes	73 (16)			
**first line therapy mIBG**	0	n.a.	no (ref.)	355 (76)	0.013	yes vs. no	0.75 (0.59 – 0.94)
			yes	114 (24)			
**first line therapy radiotherapy percutaneous**	0	n.a.	no (ref.)	408 (87)	0.089	yes vs. no	0.78 (0.58 – 1.04)
			yes	61 (13)			
**first line therapy, best result of operation**	0	n.a.	no operation	37 (8)	0.608	no operation vs. complete	0.91 (0.63 – 1.31)
			biopsy	32 (7)	0.250	biopsy vs. complete	1.25 (0.85 – 1.84)
			incomplete	194 (41)	0.008	incomplete vs. complete	0.75 (0.61 – 0.93)
			complete (ref.)	206 (44)			
**first line therapy, number of chemotherapy cycles**	0	6 (6 – 8)	n.a.		0.041		0.924 (0.857 – 0.997)
**first line therapy, number of N5 cycles**	0	3 (3 – 3)	n.a.		0.011		0.728 (0.571 – 0.930)
**first line therapy, number of N6 cycles**	0	3 (3 – 3)	n.a.		0.010		0.718 (0.559 - 0.924)

**first line therapy, number of N8 cycles**	0	0 (0 – 2)	n.a.		0.149		0.923 (0.827 – 1.029)
**first line therapy, other therapies**	0	n.a.	no (ref.)	459 (98)	0.471	yes vs. no	0.79 (0.42 – 1.49)
			yes	10 (2)			
**first line therapy, time from end to recurrence (months)**	0	1.1 (0.2 – 4.7)	n.a.		<0.001		0.98 (0.97 – 0.99)
**induction chemotherapy, time in months**	0	5.3 (4.6 – 6.6)	n.a.		0.113		0.96 (0.91 – 1.01)
**localization of tumor sites at diagnosis**	0	n.a.	only metastases (ref.)	4 (1)	0.345	PT+METS vs. METS	1.73 (0.56 – 5.38)
			primary tumor and metastases	465 (99)			
**metastasis bone/bone marrow at first diagnosis**	0	n.a.	no (ref.)	12 (3)	0.097	yes vs. no	0.62 (0.35 – 1.09)
			yes	457 (97)			
**metastasis bone/bone marrow at first recurrence**	0	n.a.	no (ref.)	157 (33)	0.236	yes vs. no	1.13 (0.92 – 1.39)
			yes	312 (67)			
**metastasis CNS at first diagnosis**	0	n.a.	no (ref.)	453 (97)	0.128	yes vs. no	1.52 (0.89 – 2.59)
			yes	16 (3)			
**metastasis CNS at first recurrence**	0	n.a.	no (ref.)	401 (85)	0.065	yes vs. no	1.29 (0.98 – 1.70)
			yes	68 (15)			
**metastasis intracranial at first diagnosis**	0	n.a.	no (ref.)	400 (85)	0.196	yes vs. no	1.20 (0.91 – 1.57)
			yes	69 (15)			
**metastasis intracranial at first recurrence**	0	n.a.	no (ref.)	453 (97)	0.030	yes vs. no	1.74 (1.05 – 2.88)
			yes	16 (3)			
**metastasis liver at first diagnosis**	0	n.a.	no (ref.)	414 (88)	<0.001	yes vs. no	1.80 (1.35 – 2.41)
			yes	55 (12)			
**metastasis liver at first recurrence**	0	n.a.	no (ref.)	420 (90)	0.007	yes vs. no	1.52 (0.12 – 2.06)
			yes	49 (10)			

**metastasis lung/pleura at first diagnosis**	0	n.a.	no (ref.)	434 (93)	0.070	yes vs. no	1.39 (0.97 – 1.99)
			yes	35 (7)			
**metastasis lung/pleura at first recurrence**	0	n.a.	no (ref.)	448 (96)	<0.001	yes vs. no	2.41 (1.55 – 3.75)
			yes	21 (4)			
**metastasis lymph nodes at first diagnosis**	0	n.a.	no (ref.)	362 (77)	0.731	yes vs. no	0.96 (0.76 – 1.21)
			yes	107 (23)			
**metastasis lymph nodes at first recurrence**	0	n.a.	no (ref.)	422 (90)	0.077	yes vs. no	1.32 (0.97 – 1.79)
			yes	47 (10)			
**metastasis other sites at first diagnosis**	0	n.a.	no (ref.)	445 (95)	0.359	yes vs. no	1.23 (0.79 – 1.91)
			yes	24 (5)			
**metastasis other sites at first recurrence**	0	n.a.	no (ref.)	457 (97)	0.337	yes vs. no	1.33 (0.75 – 2.36)
			yes	12 (3)			
**metastatic sites at first recurrence, number of**	0	1 (0 – 1)	n.a.		<0.001		1.373 (1.209 – 1.559)
**metastatic sites at first recurrence, summarized number of**	0	n.a.	none (ref.)	73 (16)			
			one	290 (62)	0.124	one vs. none	1.25 (0.94 – 1.66)
			more than one	106 (23)	<0.001	more than one vs. none	2.23 (1.62 – 3.08)
**metastatic sites, binary number of**	0	n.a.	one (ref.)	256 (55)	0.042	more than one vs. one	1.22 (1.01 – 1.48)
			more than one	213 (45)			
**metastatic sites, number of**	0	1 (1 – 2)	n.a.		0.009		1.17 (1.04 – 1.32)
**primary tumor site pelvical**	0	n.a.	no (ref.)	462 (99)	0.702	yes vs. no	1.16 (0.55 – 2.45)
			yes	7 (1)			
**primary tumor site adrenal**	0	n.a.	no (ref.)	196 (42)	0.249	yes vs. no	1.12 (0.92 – 1.36)
			yes	273 (58)			

**primary tumor site adrenal at recurrence**	0	n.a.	no (ref.)	405 (86)	0.308	yes vs. no	1.16 (0.87 – 1.53)
			yes	64 (14)			
**primary tumor site cervical**	0	n.a.	no (ref.)	461 (98)	0.394	yes vs. no	1.36 (0.67 – 2.73)
			yes	8 (2)			
**primary tumor site abdominal**	0	n.a.	no (ref.)	317 (68)	0.252	yes vs. no	0.89 (0.72 – 1.09)
			yes	152 (32)			
**primary tumor site abdominal at recurrence**	0	n.a.	no (ref.)	344 (73)	0.578	yes vs. no	0.94 (0.76 – 1.17)
			yes	125 (27)			
**primary tumor site thoracic**	0	n.a.	no (ref.)	397 (85)	0.449	yes vs. no	1.11 (0.85 – 1.44)
			yes	72 (15)			
**primary tumor site thoracic at recurrence**	0	n.a.	no (ref.)	436 (93)	0.669	yes vs. no	1.08 (0.75 – 1.56)
			yes	33 (7)			
**primary tumor sites, number of**	0	1 (1 – 1)	n.a.		0.390		1.12 (0.87 – 1.44)
**primary tumor sites, summarized number of**	0	n.a.	none (ref.)	4 (1)			
			one	427 (91)	0.353	one vs. none	1.71 (0.55 – 5.34)
			more than one	38 (8)	0.277	more than one vs. none	1.93 (0.59 – 6.28)
**recurrence at primary tumor site**	0	n.a.	no (ref.)	250 (53)	0.675	yes vs. no	1.04 (0.86 – 1.26)
			yes	219 (47)			
**recurrence sites, binary number of**	0	n.a.	one (ref.)	264 (56)	<0.001	more than one vs. one	1.71 (1.41 – 2.08)
			more than one	205 (44)			
**recurrence sites, combinations**	0	n.a.	local only (ref.)	72 (15)			
			metastases only	250 (53)	0.049	metastases only vs. local only	1.34 (1.00 – 1.78)
			local and metastases	147 (31)	0.001	local and metastases vs. local only	1.67 (1.23 – 2.27)
**recurrence sites, number of**	0	1 (1 – 2)	n.a.		<0.001		1.34 (1.20 – 1.51)

**Trial**	0	n.a.	NB97(ref.)	183 (39)	0.092	NB04 vs. NB97	1.18 (0.97 – 1.4 4)
			NB04	286 (61)			

ASCT	high-dose chemotherapy with autologous stem cell transplantation
CNS	central nervous system
CR	complete remission
d	Days
EFS	event-free survival from diagnosis to first recurrence
histology diff	histology differentiating, ganglioneuroblastoma nodular, ganglioneuroma (INPC)
histology undiff	histology undifferentiated or low differentiated (INPC)
HVA	homovanillic acid in urine at initial diagnosis
INPC	international neuroblastoma pathology classification
LDH	lactate dehydrogenase in serum at first diagnosis
LOH 1p	loss of heterozygosity of chromosome 1p
M	Months
METS	Metastases
mIBG	Metaiodobenzylguanidine
Missings*	Colors: red >15% missings, yellow >0-15% missings, green 0% missings
MR	mixed remission/stable disease
N	Number
n.a.	not applicable
NR	no remission
NSE	neuron specific enolase in serum at initial diagnosis
PR	partial remission
PROG	progression (from residual tumor)
PT	primary tumor site
REC	recurrence (from CR/VGPR)
VGPR	very good partial remission
VMA	vanillylmandelic acid in urine at initial diagnosis
Y	Years

Supplemental Table S3

Analyzed time-dependent variables with Cox proportional hazard estimations for secondary event-free survival (*N*= 469).

Variable	Missings	Categories	Numbers	Wald *P*	Comparison	HR (95% CI)
**palliative treatment**	0	yes	97	< 0.001	yes vs. no	5.324 (4.128 – 6.868)
		no (ref.)	372			
**second line therapy chemotherapy**	0	time-dependent	n.a.	< 0.001	yes vs. no (time- dependent)	0.407 (0.322 – 0.513)
**second line therapy ASCT**	0	time-dependent	n.a.	0.008	yes vs. no (time- dependent)	0.071 (0.010 – 0.504)
**second line therapy radiotherapy percutaneous**	0	time-dependent	n.a.	0.201	yes vs. no (time- dependent)	0.727 (0.446 – 1.185)
**second line therapy antibody**	0	time-dependent	n.a.	0.051	yes vs. no (time-dependent)	0.589 (0.347 – 1.001)
**second line therapy mIBG**	0	time-dependent	n.a.	0.029	yes vs. no (time- dependent)	0.212 (0.053 – 0.852)
**second line therapy operation**	0	time-dependent	n.a.	0.007	yes vs. no (time- dependent)	0.149 (0.037 – 0.599)
**second line therapy isotretinoin**	0	time-dependent	n.a.	0.576	yes vs. no (time- dependent)	0.841 (0.458 – 1.543)
**second line therapy maintenance chemotherapy**	0	time-dependent	n.a.	0.550	yes vs. no (time- dependent)	0.549 (0.077 – 3.915)

Supplemental Table S4

Distribution of prognostic index values and risk classes in the training set, validation set and total set

Risk class	PI*	Validation set (N=159) *N*	Training set (N=310) *N*	Overall set (N=469) *N*
**lower**	0.000	6	14	20
**lower**	0.253	2	12	14
**lower**	0.438	18	36	54
**intermediate**	0.570	1	1	2
**intermediate**	0.690	16	31	47
**intermediate**	0.707	5	8	13
**intermediate**	0.820	11	22	33
**intermediate**	0.822	0	1	1
**intermediate**	0.959	8	11	19
**intermediate**	1.007	2	2	4
**intermediate**	1.073	8	17	25
**intermediate**	1.144	10	18	28
**higher**	1.258	3	8	11
**higher**	1.260	2	4	6
**higher**	1.277	0	1	1
**higher**	1.390	0	5	5
**higher**	1.397	14	23	37
**higher**	1.511	5	7	12
**higher**	1.527	12	18	30
**higher**	1.529	1	1	2
**higher**	1.714	2	3	5
**higher**	1.780	11	35	46
**higher**	1.828	1	3	4
**higher**	1.965	5	6	11
**higher**	1.967	4	7	11
**higher**	2.081	1	0	1
**higher**	2.097	1	3	4
**higher**	2.218	6	8	14
**higher**	2.350	1	3	4
**higher**	2.535	3	1	4
**higher**	2.787	0	1	1

* Ranging from 0 to 2.787 with a median of 1.11 (95%-CI 1.01 - 1.14), the optimal two cut-offs were at C1=0.50 and C2=1.20.
The medians (95%-CI) for the training- and validation- set were 1.11 (1.01-1.14) and 1.14 (1.07-1.40). In both datasets, the PI was rather bimodal distributed with single peaks at the left and right side of the median.

Supplemental Table S5: Distribution of the identified risk factors in the defined risk groups.

Variable	Risk group lower (N=88) *N* (%)	Risk group Intermediate (N=172) *N* (%)	Risk group higher (N=209) *N* (%)	Overall (N=496) *N* (%)
**EFS (<18 months)**	0 (0%)	60 (34.9%)	170 (81.3%)	230 (46.4%)
**Age at diagnosis (<42 months)**	14 (15.9%)	92 (53.5%)	134 (64.1%)	240 (48.4%)
**Liver metastasis at diagnosis**	0 (0)	7 (4.1%)	48 (23.0%)	55 (11.1%)
**Progress in primary Tumor site**	34 (38.6%)	93 (54.1%)	92 (44.0%)	219 (44.2%)
**Number of recurrence sites (>1)**	0 (0)	58 (33.7%)	147 (70.3%)	205 (41.3%)

Figure S1
Kaplan-Meier estimations for secondary event-free and secondary overall survival according to the proposed risk score and with curative intent. Validation set total n=159.
Patients treated with curative intent (n=126): Risk group lower (n=24 patients), risk group intermediate (n=51 patients), and risk group higher(n=51).
Patients treated with palliative intent (n=33): Risk group lower (n=2 patients), risk group intermediate (n=10patients), and risk group higher(n=21)

Supplemental Figure S1A
EFS of patients treated with curative intent of the validation set n=126.

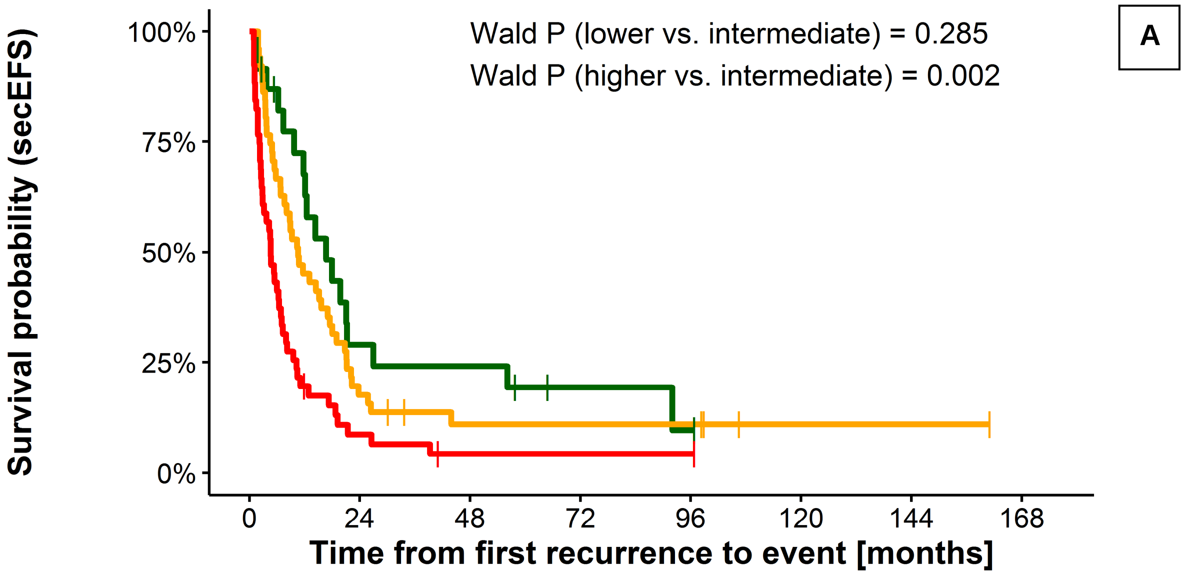

Numbers at risk

lower	24	6	5	2	1	0	0	0
intermediate	51	9	4	4	4	1	1	0
higher	51	4	1	1	1	0	0	0

The 2-year secEFS proportions were 27.8% (95%-CI 8.4 - 47.3) for risk group lower, 11.0% (95%-CI 2.0 - 20.0) for risk group intermediate, and 3.8% (95%-CI 0 - 9.7) for risk group higher.

Figure S1B

OS of patients treated in curative intent of the validation set n=126.

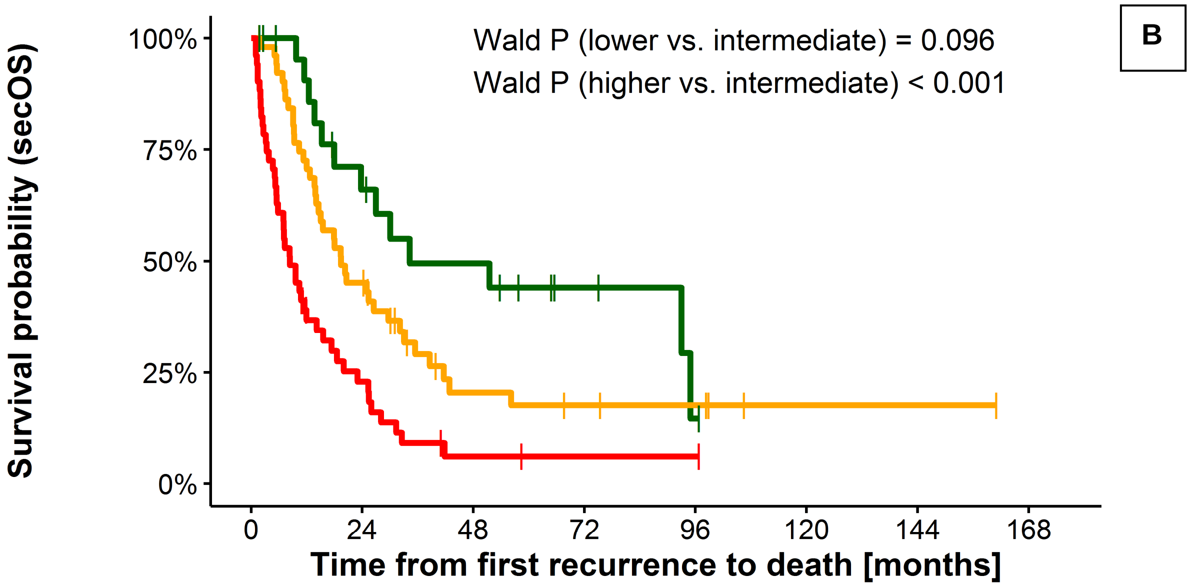

Numbers at risk

lower	24	13	9	4	1	0	0	0
intermediate	51	23	7	5	4	1	1	0
higher	51	10	2	1	1	0	0	0

The 2-year secOS proportions were 51.8% (95%-CI 29.5 – 74.2) for risk group lower, 22.6% (95%-CI 10.3 - 34.8) for risk group intermediate, and 6.1% (95%-CI 0 – 13.5) for risk group higher.

Figure S1C

EFS of patients treated in palliative intent of the validation set.

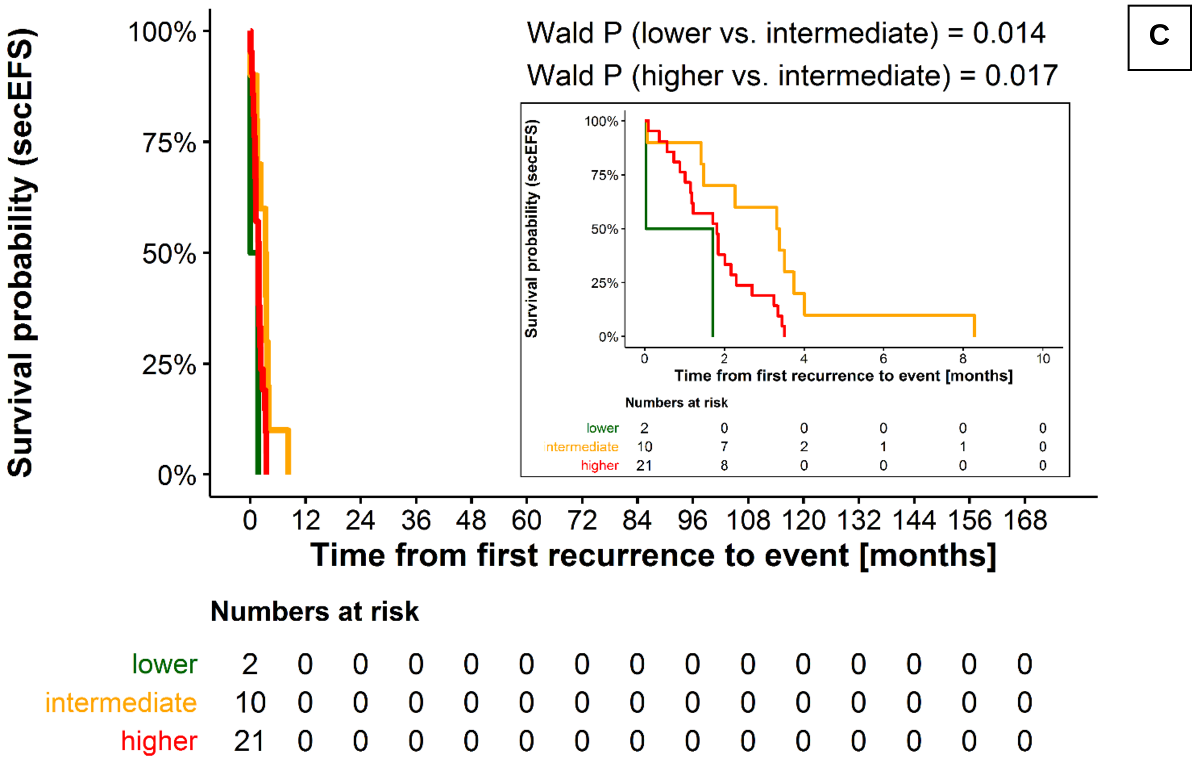

The 2-year secEFS proportions were 0% for risk group lower, 0% for risk group intermediate, and 0% for risk group higher.

Figure S1D

OS of patients treated in palliative intent of the validation set.

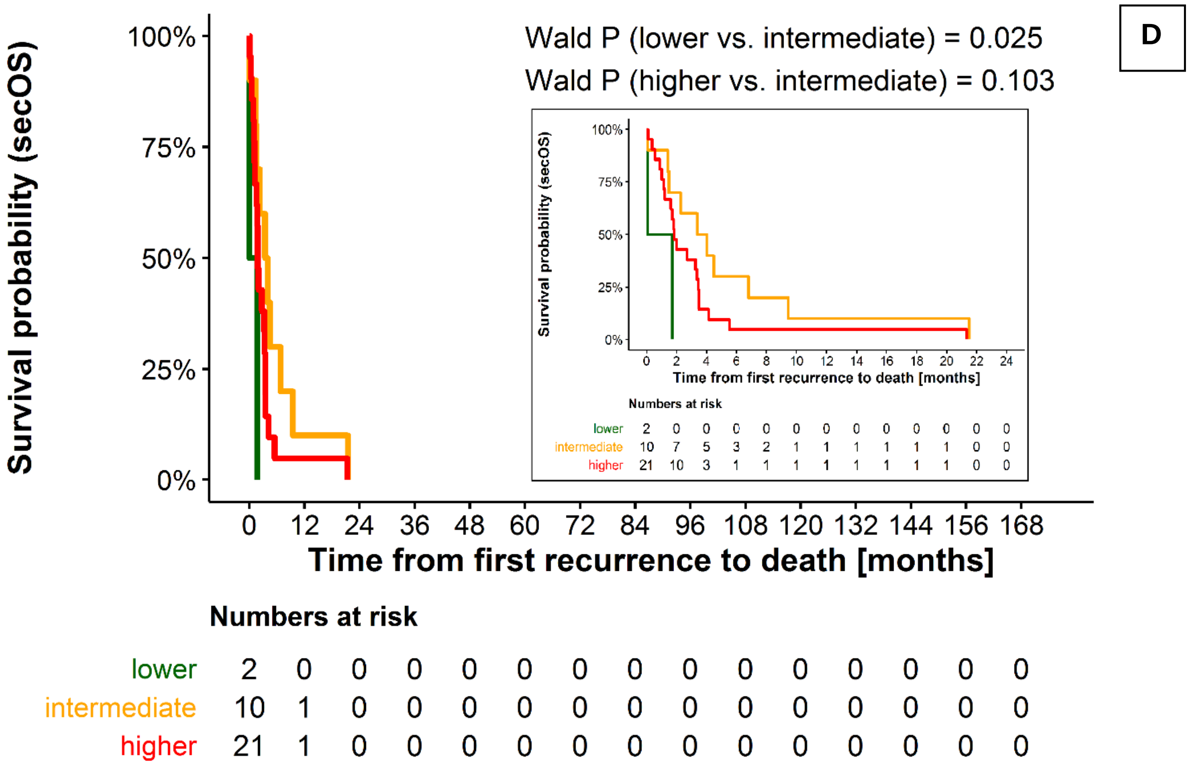

The 2-year secEFS proportions were 0 % for risk group lower, 0% for risk group intermediate, and 0% for risk group higher.
